# The effectiveness of cultural competence education in enhancing knowledge acquisition, performance, attitudes, and student satisfaction among undergraduate health science students: a scoping review

**DOI:** 10.3352/jeehp.2021.18.3

**Published:** 2021-02-24

**Authors:** Elio Arruzza, Minh Chau

**Affiliations:** UniSA Allied Health & Human Performance, University of South Australia, Adelaide, Australia; Hallym University, Korea

**Keywords:** Attitude, Cultural competency, Data management, PubMed, University students

## Abstract

**Purpose:**

Cultural competence in healthcare assists in the delivery of culturally sensitive and high-quality services. This scoping review aims to provide an overview of the available evidence and to examine the effectiveness of classroom-based intervention strategies used to enhance the cultural competence of undergraduate health science students.

**Methods:**

A comprehensive and systematic literature search was undertaken in databases, including Cochrane Library, Medline, and Emcare. Articles were eligible if they employed an experimental study design to assess classroom-based cultural competency education for university students across the health science disciplines. Two reviewers independently screened and extracted relevant data pertaining to study and participant characteristics using a charting table. The outcomes included knowledge, attitudes, skills, and perceived benefits.

**Results:**

Ten studies were analysed. Diverse approaches to cultural education exist in terms of the mode, frequency, and duration of interventions. For the knowledge outcome, students who experienced cultural education interventions yielded higher post-test scores than their baseline cultural knowledge, but without a significant difference from the scores of students who did not receive interventions. Data relating to the skills domain demonstrated positive effects for students after experiencing interventions. Overall, students were satisfied with their experiences and demonstrated improvements in confidence and attitudes towards culturally competent practice.

**Conclusion:**

Across health science disciplines, cultural competency interventions were shown to be effective in enhancing knowledge acquisition, performance of skills, attitudes, and student satisfaction. Future research is necessary to address the significant absence of control arms in the current literature, and to assess long-term effects and patient-related outcomes.

## Introduction

### Background/rationale

Cultural competence is a foundational pillar of healthcare that endeavours to reduce current disparities in delivering culturally sensitive and quality services [1]. Fundamentally, it strives to provide equal access to healthcare across diverse groups and to ensure that all patients receive care according to their needs [2]. Culturally competent services can be broadly defined as those that respect diversity amongst the patient population and the socio-cultural factors that may affect their health; this includes their beliefs, behaviours, attitudes, and language [3]. As populations become increasingly diverse due to globalization and migration, health professionals are continually finding themselves servicing patients with differing cultural and linguistic needs [4].

Numerous reviews have analysed the impacts of cultural competence interventions on registered health professionals [5,6] and students in other fields including nursing and medicine [7,8]. Although cultural competence training has varied across disciplines in terms of the frequency, duration, and overall nature of educational interventions, their conclusions indicate that cultural competence education may be effective in positively influencing the capabilities of both professionals and students. These benefits have been proposed to directly mitigate health disparities caused by a variety of factors, including social and economic conditions, access issues, insurance coverage, and genetic factors [9]. Practitioners’ increased competency levels were correlated with increased treatment adherence, patient satisfaction, and information-sharing [10]. Furthermore, when cultural differences between healthcare clinicians and healthcare users are not addressed, considerable miscommunication, mistrust, dissatisfaction, and disempowerment are undesirably fostered [11].

In recent years, many institutions have opted for a cross-cultural approach that focuses on teaching more general knowledge, attitudes, and skills that are applicable to a plethora of cultural situations [11]. The health sciences are distinct from many disciplines in that undergraduates learn theoretical knowledge through conventional didactic courses, whilst partaking in clinical placements where experience with real-world principles is continually evaluated and their professional identities are developed [12]. Education concerning cultural competence prior to these latter environments may play a key role in improving students’ understanding and equipping them with greater competence after graduation. As characterized by the majority of related studies, the dimensions of competence have generally encompassed knowledge (i.e., acquisition of cultural-related information), attitudes (i.e., beliefs and tendencies), skills (i.e., performance of cultural-related activities), satisfaction, and perceived confidence [13]. To the best of our knowledge, however, no previous review has been undertaken with an exclusive focus on undergraduate health science students.

Surveys have suggested that current healthcare workers remain unaware of the actual impact of health disparities nationwide [14]. This may be due to a deficiency in effective cultural competency in their undergraduate education, because facilitating a comprehensive curriculum that includes the entire scope of cultural competency is still a recognized challenge. Examining the approaches used to facilitate cultural competency education may help improve culturally appropriate care.

### Objectives

Therefore, this scoping review aimed to examine the intervention strategies utilized by studies and their outcomes in order to determine educational approaches that may enhance the cultural competence of undergraduate health science students. Ultimately, the findings of this study are hoped to build awareness of such education and inform the future implementation of educational research to enhance the cultural competence of graduating health professionals. Specifically, this review may aid in facilitating curricular changes that have the potential to translate into patient-related outcomes, reduce bias, and provide high-quality healthcare for all people.

## Methods

### Ethics statement

Written informed consent and ethical approval were not required due to the nature of the study.

### Reporting guideline

The Preferred Reporting Items for Systematic Reviews and Meta-Analyses Extension for Scoping Reviews (PRISMA-ScR) statement was utilized to perform this scoping review. The checklist contains 20 required items and 2 optional items for the transparent reporting of essential study aspects [15].

### Eligibility criteria

This review encompassed a wide range of both experimental and quasi-experimental study designs, including but not limited to randomized controlled trials, non-randomized controlled trials, pre-post studies, and observational studies such as prospective and retrospective cohort studies, cross-sectional studies, and case-control studies. Clinical trials, previous meta-analyses/reviews, editorial comments, and opinion pieces were excluded. Participants included undergraduate students within the health science discipline. Studies that included primary, secondary, or post-graduate students were excluded, as well as professionals in the field with existing accreditation. There exists no established definition that specifically defines the disciplines under the “health science” banner. We therefore chose to selectively include the fields that constitute the Australian National Registration and Accreditation Scheme [16] and comprise the overwhelming majority of health science and allied health clinical activity [9]. Specifically, we included undergraduate students enrolled in health science, pharmacy, physiotherapy, podiatry, medical radiation, optometry, speech pathology, and occupational therapy.

### Information sources

The Cochrane Library, Medline, and Emcare databases were systematically searched for literature published from database inception until June 2020.

### Search

The search strategy was defined through the principles of a systematic search, using the PICO (population, intervention, comparison, outcome) scheme. The search terms included the following keywords: [‘cultural competen*’ or ‘cultural awareness*’ or ‘intercultural education’ or ‘cross-cultural education’ or ‘indigenous education’] AND [‘higher education’ or ‘tertiary education’ or ‘universit*’ or ‘colleg*’ or ‘further education’ or ‘undergraduate*’] AND [‘allied health’ or ‘health’ or ‘health science*’ or ‘radiograph*’ or ‘physio*’ or ‘podiat*’ or ‘occupational therap*’ or ‘physical therap*’ or ‘speech patho*’ or ‘optometr*’ or ‘pharm*’ or ‘speech path*’ or ‘medical imag*’ or ‘medical rad*’]. The search was limited to English studies and those with human participants.

### Selection of sources of evidence

The reference lists of previous systematic reviews were investigated to find eligible studies not discovered from the systematic search. The concept of interest was classroom-based cultural competency programs administered to health science students. These programs may be elective courses or courses forming part of the compulsory university curriculum. This implies that the included studies may have provided cultural competency education via a range of delivery methods. In terms of setting, the search encompassed initiatives performed at universities, skills laboratories, or virtual classrooms, and included (but was not limited to) didactic lecture formats, tutorials, computer-based training, and simulation methods or virtual reality software. However, clinical placements, immersion experiences, and service-learning courses were excluded. No stipulation was made in regard to the duration or frequency of the educational program, implying that interventions could range from a single sitting to longer-term cultural interventions. No geographical limitation was applied.

### Data charting process (data extraction)

Two reviewers (E.S.A. and M.C.) independently extracted relevant data from the included studies. This information encompassed the following aspects: the characteristics of studies (publication year, sample size, country, field of education), participant characteristics (age and gender where possible) and characteristics relating to the intervention, control, and outcome measures (i.e., frequency and duration, measurement tools). Any disagreements that arose between the reviewers were arbitrated by consensus. When required, authors of studies were contacted to request missing or additional data.

### Data items (variables)

Articles were included if they featured any independent variable relating to the following: knowledge (i.e., acquisition of theoretical concepts), skills (i.e., practical application), self-perceived benefit (i.e., satisfaction, motivation, confidence, etc.), attitudes (i.e., beliefs and tendencies) and/or costs.

## Results

### Selection of sources of evidence

**Fig. 1.**
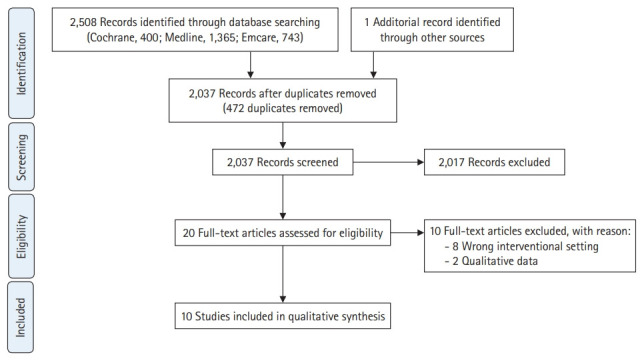
Adapted PRISMA (Preferred Reporting Items for Systematic Reviews and Meta-Analyses) flowchart.

A total of 2,508 studies were discovered through an electronic literature search, including 942 from Medline, 731 from Emcare, and 363 from the Cochrane Library. After hand-searching previous relevant systematic reviews, a further study was added. Using EndNote X9 software (Clarivate, Philadelphia, PA, USA), 472 duplicate articles were removed. The full texts of 20 articles were analysed after 2 reviewers (E.S.A. and M.C.) independently assessed the titles and abstracts of all studies found in the primary search; 2,017 articles were excluded, primarily because their topics and/or outcomes were irrelevant to the scope of our study. “Pearl growing” was undertaken by reviewing the reference lists of these selected studies for additional references unidentified in the primary search. Discussions were undertaken to resolve discrepancies between reviewers. A further 10 studies were removed after full-text analysis, due to having a qualitative study design (n=2) or not employing a classroom-based intervention (n=8). Consequently, 10 studies were included in this review. Fig. 1 presents a summary of the search and screening method, as adapted from the PRISMA statement.

### Characteristics of the sources of evidence

The characteristics of all included studies are summarised in Table 1. Aside from 1 included study conducted in 2004 [17], the publication dates spanned from 2012 until the most recent in 2019. The majority of studies were conducted in the United States (n=7) [17-23], with articles from Australia (n=2) [24,25] and Canada (n=1) [26] comprising the remainder. Eight studies employed a pre-post-test study design [17-20,22-24,26], whilst 2 simply tested participants post-intervention [21,25]. Eight of the 10 studies utilised a multimodal approach in their intervention group, which entailed a combination of any 2 or more of the following: didactic lectures, workshops, tutorials, discussion groups, case studies, student-patient interviews, and interactive activities [17,20-22,25,26]. Four of the studies featured a control group that did not experience the cultural competency intervention [19,20,22,24]. One study featured 3 groups and compared cultural competency interventions [23]. None of the studies with a control group employed blinding [19,20,22,24], though it should be noted that effective blinding is largely inconceivable in this context. The amalgamated total of participants across the studies was 1,626, with individual sample sizes ranging from 27 to 745. Articles focusing on pharmacy students comprised the most studies (n=5) [17-19,21,23], whilst the remaining related to students in occupational therapy [20,26] (n=2), podiatry [22], health science [25], and physical therapy [24] (n=1). The length of engagement varied across studies, ranging from 10 minutes [24] to the entire length of the degree (3 years) [20]. Five studies implemented their educational methods in a single “one-off” sitting [17,21,23,24,26]. Three studies reported the participation of representatives (e.g., advisors, instructors, and curriculum designers) sourced from the ethnicity of interest in the competency program [22,25,26]. Baseline characteristics were reported in 3 of the 4 control arm studies [19,22,24], albeit without P-values reporting the degree of similarity. However, their characteristics were either stated to be similar, or as in Boggis [20], analysis of co-variance was performed to adjust for differences in the initial developmental pretest.

### Results of individual sources of evidence

#### Knowledge

Five studies reported a measure regarding the acquisition of cultural knowledge [18,19,21-23]. This was often determined via a pre- and post-intervention test. The tools were largely formulated by the educators themselves, although established measurement tools, such as the Comprehensive State Empathy Scale or the Intercultural Developmental Inventory were implemented by Ward et al. [24] and Boggis [20], respectively. In the studies without a control group, each demonstrated an improvement in knowledge post-intervention. No study reported decreased or stagnant knowledge in the intervention group. In all 3 studies with control arms, though intervention students yielded higher post-test scores compared to their baseline cultural knowledge, this acquisition was not significantly higher than the improvement in the control group.

#### Perceived benefit

The term “perceived benefit” has been utilized to encompass any outcome based upon self-assessment or self-reflection as conducted by the students themselves; reported outcomes pertaining to this term included confidence [17,19], satisfaction [21,24,25], and perceived knowledge [26]. Furthermore, the study by Sales et al. [23] employed a survey that assessed participants’ ability to perform in 6 competency domains, based on their own perceptions. The three interventions featured in this study presented mixed findings based on the domain surveyed; simulation-based activities yielded positive changes in skills, case-scenarios produced desirable findings regarding awareness, and the lecture group exhibited improvements in both empathy and skills. This is concordant with Prescott and Nobel [21], who found that didactic lectures were a satisfying learning exercise for undergraduates, and more preferred than active-learning exercises.

Satisfaction was deemed “high” or “valuable” in 2 of the 3 studies reporting this variable [24,25]. In the remaining study, students were highly satisfied with the lecture component of the intervention, but less so with self-reflection activities [21]. Both studies reporting confidence adopted multiple reflection items that comprised a wider confidence-based outcome measure, making the holistic interpretation of findings more complex. For instance, Arif et al. [19] in 2019 found that students who received the intervention grew in confidence regarding their disease-state knowledge (P<0.05), cultural knowledge (P<0.05), and use of instruments (P<0.5), but their confidence in counselling patients from different cultural backgrounds did not differ significantly from the control. Assemi et al. [17] found that all items within their confidence survey demonstrated significant findings favouring competency education (P<0.001).

#### Attitudes

The effect on participants’ attitudes was reported in 4 studies [18,22,24,26]. The 2 studies featuring control arms discovered that although intervention students experienced a mean attitudinal change, there was no significant difference compared with the control group [22,24]. In the studies with no control arm, the majority of participants experienced a positive change in attitude [18,26].

#### Skills

Two studies featured skills-based outcomes [19,21]. The study by Prescott and Nobel [21] saw students achieve a skill score of 92.6% post intervention, though a pre-test and control group were absent. Arif et al. [19] in 2019 discovered that clinical skills were better amongst students who completed the elective course. To a greater magnitude than those in the control group (P<0.05), students aligned with patients’ specific health preferences and inquired about patients’ health beliefs during patient encounters.

One study employed an outcome measure titled “cultural orientation,” which holistically assessed the “critical elements of attitude, knowledge and skill development.” This approach aimed to identify improvement in orientations that ranged from “monocultural” and “transitional” mindsets, to more “global” mindsets. The intervention group trended toward, but not significantly, higher overall developmental scores than the control group (t=1.77, P=0.08).

## Discussion

### Summary of evidence (interpretation)

This review examined the effectiveness of cultural competency educational interventions in the context of pupils within various health science disciplines. Across the health science disciplines, there is significant evidence suggesting that cultural competency education positively impacts the knowledge, skills, attitudes, and self-perceived benefits of undergraduate students. Ultimately, the gathered evidence demonstrates that students who participate in cultural competency interventions gain a better understanding of cultural concepts than the competency they originally possessed. Likewise, it is rational to infer that positive findings in these academic-related outcomes should translate into patient-related outcomes, considering that health practitioners who are more skilful at their jobs, knowledgeable about their patients’ perspective, and hold positive attitudes toward their work are more likely to provide superior healthcare.

Educators in the majority of studies opted for a multimodal delivery of the cultural competency curriculum; this demonstrated positive results, particularly in the perceived benefits domain. This may suggest that healthcare educators are dedicated to maintaining a wide-ranging approach to increase interest and engagement amongst students, or that a lack of consensus exists regarding the most effective method, as highlighted by Brottman et al. [27]. The within-subject comparison of improvement between educational modes is an important undertaking to determine whether it is the delivery method, rather than the content itself, that determines knowledge retention and attitudinal change. Prescott and Nobel [21] found that 2 activities designed for student self-refection, which were hypothesized to be more captivating than traditional didactic lectures, were less preferred than those interventions. Their findings are concordant with an earlier study by Sales et al. [23] which found lectures to be impactful for students, suggesting that traditional means of delivery may still hold value in today’s growing technological landscape. Gaining feedback from students will be crucial in determining students’ ideal learning methods and environments, although feedback should be supplemented with an objective measure to determine whether a link exists between satisfaction and effectiveness of specific educational methods.

Many educators employed a one-off cultural intervention, whilst the frequency and duration of interventions varied greatly among the included studies. Although the local context of these studies may imply that this is a positive step forward, these steps are likely insufficient to develop long-term attitudes and behavioural change post-graduation. Educators and students alike must approach this form of education with a firm belief that sustaining cultural competency is a lifelong process, meaning that proficiency is impossible even after years in clinical practice, let alone after the completion of an undergraduate degree [28]. Generally, these studies demonstrated favourable results compared to those which featured longer-term and/or more frequent interventions. Although these findings may suggest that competence education is only effective in the short-term, they may be more representative of the fact that longer-term studies were more likely to have a control group.

### Comparison with previous studies

Delivery of content by educational providers and/or collaboration with people pertaining to the ethnicity or culture of focus was implemented in 3 studies [22,25,26]. These studies exhibited positive findings, reinforcing the notion that collaboration can offer prescriptive advice from relevant stakeholders about what to do and what not to do in clinical encounters. Though some may argue that these initiatives limit competency development due to excessive specificity, there are instances whereby education based on a specific culture is effective and should be promoted [28]. This may include rural areas where a large magnitude of the population is indigenous, for example, and are therefore subject to healthcare disparities compared to the remaining non-indigenous population. In these cases, it is still imperative that coordinators from the culture of focus have extensive experience working and teaching interculturally within health sciences, as well as a sophisticated theoretical understanding of cultural pedagogy.

To make interventions applicable for their students, educators are forced to search widely for educational models tailored to their study’s requirements, or simply utilize frameworks of their own. The local context within which an intervention takes place is influenced by a variety of factors and ultimately dictates the type of intervention used. These factors include the baseline maturity of students relating to cultural competency and other aspects of their education, previous personal experience, unconscious racial attitudes, and the patient population of interest [5]. A standardized measurement tool may be useful; however, it is imperative that such a tool preserves a sufficient amount of local applicability and subsequent versatility to be tailored towards student participants. This will aid competence levels as students graduate and most likely practice within the cities and nations where they undertook their study. Brottman et al. [27] pointed out that the choice to pursue a particular model or framework is currently not as imperative as the mere existence of any intervention to inform the curriculum. However, Kurtz et al. [29] highlighted that without a system-wide approach, culturally safe practice will continue to be viewed as anecdotal, an individual experience and not evidence-based. With more research, it is hoped that educators can gain confidence from the findings and eventually experience a shift in mindset to emphasize the use of a standardized model.

### Suggestions

A number of recommendations can be made to inform future research in the field. Studies comparing the effectiveness of differing models (e.g., didactic lectures versus class discussion, elective versus compulsory courses) would be useful for determining the most suitable educational approach. The inclusion of digital interventions would be extremely useful given the rapidity of recent technological advancements and their influence on pedagogy. Data regarding costs of implementation and qualitative discussion concerning accessibility of resources would be advantageous in enabling financial and resource analyses of specific interventions. It is hoped that students experiencing university-based competence education are then followed up during their graduate careers as they actively incorporate lessons learnt into clinical practice. This will aid in the determination of whether undergraduate education achieves its overarching goal of providing culturally sensitive healthcare, by providing avenues for researchers to pursue more relevant outcomes such as patient benefits and/or adverse effects. After all, cultural competence education may only be deemed beneficial in the long-term if it is perceived to be so by the “end-user.”

### Limitations

This scoping review only included English-language studies, meaning that research published in other languages was not analysed. There always exists the possibility that the systematic search did not acquire all relevant literature as per the inclusion criteria. Furthermore, many institutions may be conducting cultural intervention without reporting it; some of the eligible health science disciplines were not represented in the final cohort of studies, and only studies from 3 nations were included for analysis. The likelihood of publication of studies with positive findings is greater than that of studies presenting undesirable ones, implying that the literature available may overvalue the true effectiveness of cultural competency interventions. Real-world outcomes such as costs were not reported in any included study. The methodological quality of studies should be improved where possible by including a control arm with matched baseline characteristics.

## Conclusion

Cultural competence in healthcare ensures the delivery of culturally sensitive and quality services. Therefore, cultural competence has become a mainstream education issue applicable to all health science students. Our scoping review shows that cultural competency education could positively improve key student outcomes such as acquired knowledge, skills, satisfaction, confidence, and attributes. Students who participate in cultural competency interventions in their undergraduate studies gain a better understanding of cultural concepts. However, there exists a deficiency in research regarding the variable of time on cultural competency education. With regards to the cultural competency curriculum, health science educators often use multimodal delivery, for which positive results in the perceived benefits domain have been strongly demonstrated. Given rapid advancements in technology and pedagogy, it is recommended that digital interventions could prove useful in the future cultural competency curriculum. Moreover, longitudinal research following health science students is required to ascertain whether undergraduate cultural competency interventions could achieve the overarching goal of providing culturally sensitive healthcare.

## Figures and Tables

**Table 1.** Characteristics and key findings of included studies

Study	Country	Field	Design	Intervention	No. of participants	Frequency	Duration	Control	No. of participants	Outcome (outcome measure)	Key findings
Arif et al. [18] (2017)	USA	Pharmacy	One-group pre-post test	Cross-cultural communication training added to a healthcare communications course	159	Weekly	6 wk (3 hr sessions)	-	-	Knowledge (9 MCQ survey)	Students experienced an increase in knowledge (pre=5.5 vs. post=6.6, P<0.05) and attitudes (P<0.002).
				Lectures and workshops						Attitudes (Likert-scale survey)	
Arif et al. [19] (2019)	USA	Pharmacy	Two-group pre-post	Health Promotion and Disease Prevention Across Cultures course	31	Weekly	6 wk (1 hr sessions)	No intervention	31	Knowledge (12Q quiz)	Intervention participants experienced a significant increase in knowledge (pre=66.4% vs. post=74.2%, P=0.03).
				Lectures, workshops sessions, simulations, and community health-screening event						Skills (6-item evaluation rubric)	Intervention participants yielded slightly higher knowledge scores than control group students (74.2% vs. 68.3%) with no significant difference (P=0.07).
										Confidence (13Q self-assessment)	Clinical skills were better among students who completed the elective-course (P<0.05).
											Intervention students grew in confidence regarding cultural health practice (P<0.05).
Assemi et al. [17] (2004)	USA	Pharmacy	One-group pre-post test	Cultural Competency in Pharmaceutical Care	58	Once	8 hr	-	-	Confidence (12Q survey [5-point scale])	All items significantly improved post-intervention (P<0.001).
				Didactic lecture, class discussions, self-reflective exercises, and role play							
Boggis [20] (2012)	USA	Occupational therapy	Two-group pre-post test	Curricular program guided by the Intercultural Developmental Continuum	17	-	3 yr	Competency program *not* guided by an intercultural mode	25	Cultural orientation (50Q [5-point scale] Intercultural Developmental Inventory)	Intervention students demonstrated a non-significant change in overall developmental orientation mean scores (t=0.847, P=0.41).
Jamieson et al. [26] (2017)	Canada	Occupational therapy	One-group pre-post test	Aboriginal Cultural Safety Initiatives modules in Socio-Cultural Determinants of Occupation course	27	Once	3 hr	-	-	Perceived benefit (5Q survey [5-point scale])	The majority of participants (74.1%–92.6% depending on the item) showed scores suggesting improvement in perceived knowledge.
				Didactic teaching, story sharing, interactive activities, and reflective discussions						Attitudes (3Q survey [5-point scale])	The majority of students (55.6%–63.0% depending on the item) perceived an increase in their cultural/emotional responses.
Kickett et al. [25] (2014)	Australia	Health science	One-group post-test	Indigenous Cultures & Health course	745	Weekly	12 wk (2 hr sessions)	-	-	Satisfaction (13Q survey)	Overall satisfaction for first cohort (n=147) was 94% and for second cohort (n=598) was 76%.
				Video podcasts, group presentations, and class discussions							
Prescott & Nobel [21] (2019)	USA	Pharmacy	One-group post-test	Cultural competency education within Pharmaceutical Care I course	136	Once	L: 60 min; A: 30 min; P: 60 min	-	-	Knowledge (9MCQ quiz)	Students scored an average of 86.1% on the in-class quiz and 92.6% on the practicum.
				Didactic lecture (L), active-learning exercises (A) & practicum (P)						Skills (7Q reflection)	The practicum and lecture were more preferred than active-learning exercises.
										Satisfaction (11Q survey)	
Sales et al. [23] (2013)	USA	Pharmacy	Three-group pre-post test	Written case scenario activity	36	Once	1 hr	Lecture	36	Perceived benefit (15Q survey [5-point scale])	Overall, cultural competency was not significantly enhanced by any of the 3 interventions (P>0.05).
				Simulated patient encounter activity							Each intervention demonstrated improvement in at least 1 (of 6) cultural competency domains.
Smith et al. [22] (2016)	USA	Podiatry	Two-group pre-post test	Cultural Competency course	42	Weekly	10 wk	No intervention	37	Knowledge (21 MCQ)	Intervention participants experienced an improvement in mean knowledge acquisition scores of 4.71 points and increased mean attitudinal change by 2.4 points (P<0.001).
				Videos, online group discussions, and self-reflective essay						Attitudes (4Q [4-point scale])	However, no significant difference was exhibited relative to the control group (P>0.05).
Ward et al. [24] (2018)	Australia	Physical therapy	Two-group pre-post test	Virtual cultural simulation experience	162	Once	10 min	No intervention	84	Empathy (30Q CSES)	Empathy improved after simulation, shown in overall CSES scores (pre=95 [81–109] vs. post=106 [89–117]; median difference=11; P<0.001).
										Satisfaction (8Q SCSES)	Satisfaction was reported to be “high” (mean SCSES=71%).
										Attitudes (30Q TPB:CCQ)	Scores were not significantly different between both groups (4.57±1.14 vs. 4.60±1.23, P=0.45).

MCQ, multiple choice question; Q, question; CSES, Comprehensive State Empathy Scale; SCSES, Satisfaction with Cultural Simulation Experience Scale; TPB:CCQ, Theory of Planned Behaviour:Cultural Competence Questionnaire.

## Supplementary materials

Supplement 1. Audio recording of the abstract.
